# Supportive interactions with primary care doctors are associated with better mental health among transgender people: results of a nationwide survey in Aotearoa/New Zealand

**DOI:** 10.1093/fampra/cmac005

**Published:** 2022-03-09

**Authors:** Gareth J Treharne, Rona Carroll, Kyle K H Tan, Jaimie F Veale

**Affiliations:** Department of Psychology, University of Otago, Dunedin, Aotearoa, New Zealand; Department of Primary Health Care and General Practice, University of Otago, Wellington, Aotearoa, New Zealand; Trans Health Research Lab, School of Psychology, University of Waikato, Hamilton, Aotearoa, New Zealand; Trans Health Research Lab, School of Psychology, University of Waikato, Hamilton, Aotearoa, New Zealand

**Keywords:** depression/mood disorder, doctor–patient relationship, gay, lesbian, bisexual, transgender (GLBT) issues, gender, LGBTQ, physician competency, primary care

## Abstract

**Background:**

Past research has established that transgender people experience significant disparities in mental health outcomes and healthcare dissatisfaction compared with cisgender people, but more research is needed on how supportive healthcare interactions relate to the mental health of transgender people.

**Objectives:**

The 2 main aims of our analyses were: (i) to establish the most common negative experiences in healthcare and the most common supportive experiences specifically with primary care doctors for transgender people; and (ii) to examine the association of supportive experiences with mental health variables after controlling for demographic factors.

**Methods:**

Data from the 2018 *Counting Ourselves* nationwide survey of transgender people were analysed using regression modelling. The 948 participants with a primary care doctor or general practitioner were included in analyses. Participants were aged 14–83 years old (mean 30.20).

**Results:**

The most common supportive experiences involved primary care doctors treating transgender people equitably, with competence, and with respect. Participants with more negative healthcare experiences had higher psychological distress as well as higher likelihood of reporting nonsuicidal self-injury and suicidality. Conversely, participants with more experiences of supportive primary care doctors had lower psychological distress and were less likely to have attempted suicide in the past 12 months.

**Conclusion:**

When transgender people receive supportive care from their primary care providers they experience better mental health, despite ongoing negative healthcare experiences. Future research is needed to confirm ways of supporting positive trajectories of mental health for transgender people but these findings demonstrate the importance of positive aspects of care.

**Key messages**

- Transgender people experience disparities in mental health that require research.
- We analysed data from the *Counting Ourselves* survey of 948 transgender people.
- Around half of transgender people reported supportive primary care experiences.
- Supportive experiences correlated with lower distress and lower suicidality.
- These findings highlight changes needed to improve care for transgender people.
- Medical education and continuing education on transgender competency are needed.

## Introduction

Negative primary care experiences can perpetuate mental health disparities for transgender people.^1–3^ The term transgender refers to people whose gender differs from cisnormative expectations that there are only 2 genders (i.e. women and men), which are normatively expected to align with sex assigned at birth.^4–6^ In contrast, cisgender people experience privilege because their gender meets normative expectations for their assigned sex. There is a pressing need to understand the primary care experiences of transgender people and how these experiences relate to mental health.^4^

A population-based survey, the 2020 Household Economics Survey, reported 0.8% adults aged 18 or above identified as transgender and/or nonbinary in Aotearoa/New Zealand (the location of the present study).^7^ In addition, around 1.0%–1.2% of high school students in the Youth2000 survey series in Aotearoa/New Zealand reported being transgender (including nonbinary identities).^8,9^ All primary care health professionals increasingly need the knowledge and skills to create supportive interactions with transgender people, particularly with young people as they come to know their gender.

Surveys in Aotearoa/New Zealand show high rates of depression, suicidality, and self-harm amongst transgender people.^9–12^ In the Youth’19 survey, 57% of transgender students experienced depression, 57% reported nonsuicidal self-injury (NSSI), and 26% had attempted suicide in the past 12 months.^8^ Moreover, 55% of transgender youth reported being unable to access healthcare when they had needed it in the past 12 months.^8^ Transgender people have the right to the highest attainable level of health yet continue to face barriers in accessing primary care even in relatively inclusive countries like Aotearoa/New Zealand.^13^

Health professionals often report discomfort and lack of confidence providing care to transgender patients,^14,15^ and education on this topic is lacking in medical schools.^16–18^ A 2018 survey of medical school curriculum leaders in Aotearoa/New Zealand found that the majority included very little or no content about sexuality or gender identity.^16^ Since then there have been efforts by individual departments within medical school to include teaching related to gender identity, but it is not a standard learning outcome in medical school curricula in Aotearoa/New Zealand at this time.^19,20^ Having specialist transgender knowledge is not necessarily a prerequisite to positive interactions in a consultation.^15,21^ Simple actions that demonstrate respect towards transgender people contribute to positive healthcare experiences.^22–24^ Unfortunately, primary care professionals commonly misgender transgender patients and use incorrect names, highlighting the immediate need to build greater transgender cultural competence.^22–24^

Research from different countries consistently shows that negative healthcare experiences among transgender people leads to foregone healthcare, and that transgender people’s comfort with their primary care doctors positively correlates with general health and mental health.^1–3^ 62.9% of respondents to the US Trans Survey reported health professionals treating them with respect after knowing they are transgender, whereas 24.3% reported having had to educate health professionals about transgender people when seeking care.^1,25^ Those who reported depression or suicidality were significantly less likely to have had health professionals treat them with respect (odds ratio [OR] 0.67 and 0.88, respectively) and significantly more likely to have needed to educate health professionals (OR 1.17 and 1.33, respectively).^1^

Whilst past research suggests positive interactions with primary care doctors are associated with better mental health outcomes for transgender people internationally,^1–3^ this association has not been researched in Aotearoa/New Zealand in the context of publicly subsidized healthcare for all people, including transgender people.^26^ Therefore, in this paper we report on data from *Counting Ourselves: Aotearoa New Zealand Trans and Non-Binary Health Survey* that allows novel investigation of the relationship of transgender people’s mental health with both positive and negative aspects of healthcare, building on the typical focus of research on negative experiences of healthcare. The research questions were: (i) What are the common negative experiences in healthcare or supportive experiences specifically with primary care doctors in Aotearoa/New Zealand? (ii) Are there differences by demographics (age, gender, ethnicity, region, or income level) in supportive experiences with primary care doctors or mental health variables (psychological distress, NSSI, or suicidality) among transgender people in Aotearoa/New Zealand? (iii) Do mental health variables correlate with supportive experiences with primary care doctors for transgender people in Aotearoa/New Zealand after controlling for relevant demographics and negative experiences in healthcare?

## Methods

### Participants

Participants completed the 2018 *Counting Ourselves* survey, a community-based survey of transgender people aged 14 years or older, residing in Aotearoa/New Zealand. Ethics approval was granted by the New Zealand Health and Disability Ethics Committee. Participants were recruited through social media advertisements and connections made with transgender and queer community groups as well as networks of academic researchers and health professionals.

The majority of participants completed the survey online and a small number completed a paper copy. A total of 1,380 participants initiated the survey, of whom 202 were removed for not meeting all inclusion criteria (see the project report for more details).^27^ Out of the 1,178 respondents meeting the inclusion criteria, 193 participants did not complete the questions on healthcare experiences. In addition, 37 participants who noted they did not have a primary care doctor were not analysed in this article.

The demographic details of the 948 included participants are presented in Table 1. Ethnicity was classified into independent categories using the Ministry of Health’s priority protocols^28^ in the order of Māori, Pacific Islander, Asian, other ethnicities (non-New Zealand European/Pākehā), and New Zealand European/Pākehā only (equivalent to White in other contexts). A high proportion of participants were young, nonbinary, New Zealand European/Pākehā, with an annual income less than $50,001, and from 1 of 2 major urban regions (Table 1).

**Table 1.** Demographic details of participants in the 2018 *Counting Ourselves* survey with a primary care doctor or GP (*N* = 948).

Variable	Mean (SD)
Age	30.20 (13.57)
	*n* (%)
Gender groups^a^	
Trans women	276 (29.2)
Trans men	273 (28.9)
Nonbinary AFAB	306 (32.3)
Nonbinary AMAB	91 (9.6)
Prioritized ethnicity groups^b^	
New Zealand European/Pākehā and others	761 (80.3)
Māori	119 (12.6)
Pacific Islander	32 (3.4)
Asian	36 (3.8)
Regions^c^	
Auckland	286 (30.7)
Wellington	262 (28.1)
Other North Island region	163 (17.5)
South Island	221 (23.8)
Income level in past 12 months^d^	
Loss or zero income	63 (7.9)
$1–15,000	284 (35.5)
$15,001–50,000	290 (36.2)
$50,001 or more	164 (20.5)

Gender groups were derived from a 2-step approach comparing self-defined gender and sex assigned at birth.^24^ Two nonbinary participants did not report their sex assigned at birth. AFAB, assigned female at birth; AMAB, assigned male at birth.

New Zealand European/Pākehā and other ethnicities were combined as most others had specific European ethnicities.^25^

Participants who did not provide a postcode were excluded (*n* = 16).

Numbers here represent those participants who had not dropped out of the survey before providing income towards the end.

### Measures

Participants were asked 2 sets of questions about healthcare experiences from the 2015 US Trans Survey.^25^

#### Negative Healthcare Experiences Index

Participants were asked “Have you ever had any of these things ever happened to you, as a trans or non-binary person, when you were trying to access healthcare? Please include experiences with any person(s) involved with your care including doctors, nurses, and administrative staff.” The 18 items are listed in Table 2 (in ranked order). The index had high internal consistency in this sample (Cronbach’s α = 0.87).

**Table 2.** Responses to the Negative Healthcare Experiences Index ranked by frequency among participants in the *Counting Ourselves* survey with a primary care doctor or GP in 2018 (*N* = 948).

Ranking	Negative Healthcare Experiences Index items	Overall	By gender^a^			
			TW	TM	NB AFAB	NB AMAB
		*n* (%)	*n* (%)	*n* (%)	*n* (%)	*n* (%)
1	You had to teach someone about trans or non-binary people so that you could get appropriate care	446 (47.0)	124 (44.9)	175 (64.1)	120 (39.2)	25 (27.5)
2	You were asked unnecessary or invasive questions about being trans or non-binary that were not related to the reason for your visit	352 (37.1)	109 (39.5)	135 (49.5)	84 (27.5)	22 (24.2)
3	You were told they don’t know enough about gender-affirming care to provide it	262 (27.6)	72 (26.1)	110 (40.3)	64 (20.9)	14 (15.4)
4	A provider knowingly referred to you by the wrong gender, either in person or in a referral	251 (26.5)	63 (22.8)	106(38.8)	68 (22.2)	13 (14.3)
5	A provider knowingly used an old name that you are no longer comfortable with	198 (20.9)	50 (18.1)	83 (30.4)	54 (17.6)	10 (11.0)
6	A provider thought the gender listed on your ID or forms was a mistake	185 (19.5)	46 (16.7)	84 (30.8)	47 (15.4)	7 (7.7)
7	A provider used hurtful or insulting language about trans or non-binary people	173 (18.2)	43 (15.6)	71 (26.0)	46 (15.0)	11 (12.1)
8	You were discouraged from exploring your gender	160 (16.9)	39 (14.1)	54 (19.8)	55 (18.0)	11(12.1)
9	You could not access an appropriate bathroom	149 (15.7)	21 (7.6)	51 (18.7)	67 (21.9)	10 (11.0)
10	You were told that you were not really trans or non-binary	138 (14.6)	37 (13.4)	47 (17.2)	42 (13.7)	11 (12.1)
11	A provider refused to discuss or address gender-affirming healthcare	135 (14.2)	39 (14.1)	49 (17.9)	38 (12.4)	8 (8.8)
12	A provider belittled or ridiculed you for being trans or non-binary	85 (9.0)	26 (9.4)	31 (11.4)	25 (8.2)	2 (2.2)
13	Refused to provide you with a referral for gender-affirming care	85 (9.0)	27 (9.8)	36 (13.2)	20 (6.5)	2 (2.2)
14	You were refused care or had care ended because you are trans or non-binary	82 (8.5)	26 (9.4)	34 (12.5)	16 (5.2)	4 (4.4)
15	A provider examined your body when you thought it was inappropriate or it was not clear why it was necessary	57 (6.0)	26 (9.4)	16 (5.9)	10 (3.3)	4 (4.4)
16	You were placed in an incorrect hospital ward for your gender	52 (5.5)	15 (5.4)	22 (8.1)	13 (4.2)	2 (2.2)
17	A provider was physically rough or abusive when treating you	43 (4.5)	12 (4.3)	17 (6.2)	10 (3.3)	3 (3.3)
18	A provider refused to examine parts of your body because you are trans or non-binary	18 (1.9)	6 (2.2)	6 (2.2)	5 (1.6)	0
		Mean (SD)	Mean (SD)	Mean (SD)	Mean (SD)	Mean (SD)
	Number of items selected	3.03 (3.57)	2.83 (3.34)	4.12 (3.83)	2.56 (3.43)	1.75 (2.81)

Two nonbinary participants did not report their sex assigned at birth. AFAB, assigned female at birth; AMAB, assigned male at birth; NB, nonbinary gender, TM, transgender men; TW, transgender women.

#### Supportive Doctors Index

Participants were asked “How have doctors (GPs) been supportive of you?” The 7 items are listed in Table 3 (in ranked order). This index also had high internal consistency in this sample (Cronbach’s α = 0.89).

**Table 3.** Responses to the Supportive Doctor Index among *Counting Ourselves* participants with a primary care doctor or GP in 2018 (*N* = 947; ranked by frequency).

Ranking	Supportive Doctor Index items	Overall	By gender^a^			
			TW	TM	NB AFAB	NB AMAB
		*n* (%)	*n* (%)	*n* (%)	*n* (%)	*n* (%)
1	Treated you the same as any other patient when your needs were not directly related to gender-affirming care	539 (56.9)	197 (71.4)	189 (69.2)	116 (38.0)	35(38.5)
2	Been supportive of your needs relating to gender-affirming care	456 (48.2)	193 (69.9)	175 (64.1)	64 (21.0)	22 (24.2)
3	Always used your current name, with you and in referrals	449 (47.4)	175 (63.4)	170 (62.3)	84 (27.5)	19 (20.9)
4	Shown they were willing to educate themselves on gender-affirming care, if necessary	403 (42.6)	155 (56.2)	158 (57.9)	70 (23.0)	19 (20.9)
5	Always used your correct gender pronouns, with you and in referrals	387 (40.9)	160 (58.0)	156 (57.1)	52 (17.0)	18(19.8)
6	Been able to clearly explain why any and all examinations were necessary	344 (36.3)	133 (48.2)	136 (49.8)	60 (19.7)	14 (15.4)
7	Shown they knew a lot about gender-affirming care	225 (23.8)	91 (33.0)	86 (31.5)	38 (12.5)	8 (8.8)
		Mean (SD)	Mean(SD)	Mean (SD)	Mean (SD)	Mean (SD)
	Number of items selected	2.96 (2.62)	4.00 (2.46)	3.92 (2.57)	1.59 (2.11)	1.48 (2.00)

Two nonbinary participants did not report their sex assigned at birth. AFAB, assigned female at birth; AMAB, assigned male at birth; NB, nonbinary gender, TM, transgender men; TW, transgender women.

#### Psychological distress

The Kessler Psychological Distress Scale (K10) was used to assess psychological distress in the last 4 weeks.^29^ K10 has 10 items with a 5-point response scale, and higher scores indicating higher psychological distress. The internal consistency of the K10 was high in this study (α = 0.94), confirming reliability.

#### Nonsuicidal self-injury

Participants were asked 1 question about NSSI from the Youth’12 study: “During the last 12 months, have you deliberately hurt yourself or done anything you knew might have harmed you (but not kill you)?” with a 5-point response scale from “not at all” to “more than 5 times.”^9^

#### Suicidality

Participants were asked 2 questions about suicidal ideation and attempts from the Youth’12 study: “In the last 12 months, have you seriously thought about killing yourself (attempting suicide)?” and “In the last 12 months, have you tried to kill yourself (attempted suicide)?” both with a response scale of “not at all,” “once or twice,” and “three times or more.”^9^

### Data analysis

The authors of the paper include some who led the *Counting Ourselves* project and others who have collaborated on analyses for this paper. Analyses were conducted in SPSS version 27. Missing values were imputed for income (13.1%) using the expectation maximization method,^30^ where values were estimated by regression methods based on means and covariances of education, income, employment status, and deprivation measures adopted from the 2016 New Zealand General Social Survey.^31^ Missing values for K10 items were also imputed for 0.2%–1.1% of the sample through the expectation maximization method of existing scale items.

Bivariate linear regressions were used to test for differences in participants’ supportive experiences with primary care doctors across age, gender, prioritized ethnicities, region, income, and negative healthcare experiences. Ethnicity was excluded as a covariate in subsequent analyses as we did not detect a statistically significant difference in level of supportive care. Next, we employed linear regression (for K10 psychological distress) and ordinal logistic regression (for NSSI, suicidal ideation, and suicide attempts) to test associations of these mental health variables with supportive experiences with primary care doctors. Covariates in multivariate models included age, gender groups, regions, income, and negative healthcare experience.

## Results

The prevalence of negative healthcare experiences and supportive experiences with primary care doctors are shown in Tables 2 and 3, respectively. Nearly half of participants reported having to educate someone in a healthcare setting to get appropriate care. Other common negative healthcare experiences included being asked unnecessary or invasive questions and healthcare professionals stating that they are not competent in transgender care. On average, participants had experienced around 3 of the 18 negative healthcare experiences (Table 2), and this was used as the index variable.

Supportive care experiences are outlined in Table 3. Just over half of participants felt that they were treated the same as other patients when they consulted primary care doctors for needs unrelated to gender-affirming care. Almost half of participants reported that their primary care doctors were supportive of their needs related to gender-affirming care and slightly fewer noted that their primary care doctors were willing to educate themselves about gender-affirming care. Similarly, almost half of participants noted their primary care doctors used their correct current name and slightly fewer used correct current pronouns. Just over a third of participants noted that their primary care doctors had been able to clearly explain why examinations were necessary, and just less than a quarter reported that their primary care doctors had shown they knew a lot about gender-affirming care. On average, participants had experienced around 3 of the 7 supportive experiences with primary care doctors (Table 3) and this was used as the index variable.

Demographic differences for total numbers of supportive experiences with primary care doctors are detailed in Table 4. Bivariate models showed significantly more positive experiences with primary care doctors were reported by (i) older participants, (ii) those from the Wellington region compared with those from Auckland, and (iii) trans women compared with nonbinary participants.

**Table 4.** Bivariate linear regression of sociodemographic differences in level of supportive experiences with primary care doctors among participants in the 2018 *Counting Ourselves* survey with a primary care doctor or GP (*N* = 947).

Variable	*B*	SE	*P* value
Age groups			<0.001
14–19	Reference		
20–24	0.73	0.25	0.004
25–34	0.70	0.24	0.003
35–44	0.78	0.31	0.012
45–54	1.12	0.35	0.002
55 and over	1.82	0.35	<0.001
Gender groups			<0.001
Trans women	Reference		
Trans men	−0.08	0.20	0.687
Nonbinary AFAB	−2.41	0.19	<0.001
Nonbinary AMAB	−2.52	0.28	<0.001
Prioritized ethnicity groups			0.197
New Zealand European and others	Reference		
Māori	0.17	0.26	0.519
Pacific Islander	−0.77	0.47	0.104
Asian	−0.54	0.45	0.225
Regions			0.017
Auckland	Reference		
Wellington	0.60	0.22	0.008
Other North Island region	0.04	0.26	0.872
South Island	−0.06	0.23	0.806
Income level in last 12 months			0.202
Loss or zero income	Reference		
$1–15,000	0.24	0.36	0.515
$15,001–50,000	0.46	0.36	0.201
$50,001 and more	0.67	0.38	0.079

Regression analyses of mental health variables and effect sizes are reported in Table 5. Higher levels of negative healthcare experiences were associated with higher levels of psychological distress and higher rates of NSSI and suicidality in bivariate models. Conversely, higher levels of supportive experiences with primary care doctors were associated with lower psychological distress and NSSI in bivariate models. In multivariate models that adjusted for demographics and healthcare experiences, participants with more negative healthcare experiences had significantly higher psychological distress as well as higher likelihood of reporting NSSI and suicidality. Participants with higher levels of supportive experiences with primary care doctors had lower psychological distress and were less likely to have attempted suicide in the past 12 months.

**Table 5.** Regression models of associations of mental health outcomes with negative healthcare experiences and support from doctors among participants in the 2018 *Counting Ourselves* survey with a primary care doctor or GP (*N* = 783).

Variable	K10		NSSI		Suicidal ideation		Suicide attempt	
	Bivariate	Multivariate	Bivariate	Multivariate	Bivariate	Multivariate	Bivariate	Multivariate
	Unadjusted *B*	Adjusted *B*	Unadjusted OR	Adjusted OR	Unadjusted OR	Adjusted OR	Unadjusted OR	Adjusted OR
Age	−0.31 [−0.35 to −0.27]^∗∗^	−0.29 [−0.34 to −0.25]^∗∗^	0.92 [0.91 to 0.94]^∗∗^	0.93 [0.91 to 0.95]^∗∗^	0.96 [0.95 to 0.97]^∗∗^	0.97 [0.96 to 0.98]^∗∗^	0.95 [0.93 to 0.97]^∗∗^	0.97 [0.93 to 1.00]^∗^
Gender groups								
Trans women	Reference	Reference	Reference	Reference	Reference	Reference	Reference	Reference
Trans men	3.76 [2.13 to 5.38]^∗∗^	−0.47 [−2.07 to 1.14]	2.45 [1.73 to 3.48]^∗∗^	1.16 [0.78 to 1.74]	1.58 [1.13 to 2.19]^∗^	0.93 [0.64 to 1.36]	1.84 [1.06 to 3.14]^∗^	1.13 [0.60 to 2.17]
Nonbinary AFAB	4.72 [3.14 to 6.30]^∗∗^	0.53 [−1.13 to 2.19]	1.98 [1.40 to 2.78]^∗∗^	0.98 [0.64 to 1.49]	1.15 [0.84 to 1.58]	0.79 [0.53 to 1.17]^∗^	1.04 [0.59 to 1.86]	0.56 [0.28 to 1.13]
Nonbinary AMAB	−0.71 [−3.06 to 1.65]	−1.56 [−3.84 to 0.73]	0.90 [0.52 to 1.57]	1.04 [0.54 to 1.96]	0.56 [0.34 to 0.92]^∗^	0.67 [0.38 to 1.16]^∗^	0.49 [0.14 to 1.32]	0.50 [0.11 to 1.65]
Regions								
Auckland	Reference	Reference	Reference	Reference	Reference	Reference	Reference	Reference
Wellington	−0.46 [−2.13 to 1.21]	−0.93 [−2.43 to 0.57]	1.11 [0.78 to 1.58]	1.04 [0.70 to 1.55]	0.82 [0.59 to 1.14]	0.72 [0.50 to 1.03]	0.78 [0.44 to 1.41]	0.94 [0.49 to 1.80]
Other North Island region	1.58 [−0.35 to 3.51]	1.26 [−0.45 to 2.97]	1.36 [0.92 to 2.02]	1.26 [0.82 to 1.95]	0.92 [0.63 to 1.35]	0.84 [0.56 to 1.26]	0.80 [0.40 to 1.60]	0.65 [0.29 to 1.38]
South Island	2.39 [0.63 to 4.14]^∗^	1.76 [0.19 to 3.34]^∗^	1.67 [1.17 to 2.39]^∗^	1.56 [1.04 to 2.32]^∗^	1.20 [0.85 to 1.69]	1.15 [0.79 to 1.66]	1.30 [0.74 to 2.28]	1.52 [0.81 to 2.88]
Income level	−3.93 [−4.62 to −3.25]^∗∗^	−1.85 [−2.59 to −1.10]^∗∗^	0.49 [0.42 to 0.57]^∗∗^	0.79 [0.65 to 0.96]^∗^	0.60 [0.52 to 0.69]^∗∗^	0.76 [0.64 to 0.91]^∗∗^	0.47 [0.36 to 0.62]^∗∗^	0.61 [0.44 to 0.85]^∗^
Negative Healthcare Experience Index	0.54 [0.36 to 0.71]^∗∗^	0.48 [0.32 to 0.65]^∗∗^	1.08 [1.04 to 1.12]^∗∗^	1.08 [1.04 to 1.13]^∗∗^	1.11 [1.07 to 1.16]^∗∗^	1.09 [1.05 to 1.14]^∗∗^	1.18 [1.12 to 1.24]^∗∗^	1.20 [1.13 to 1.27]^∗∗^
Supportive Doctor Index	−0.70 [−.94 to −0.46]^∗∗^	−0.50 [−0.74 to −0.24]^∗∗^	0.93 [0.88 to 0.98]^∗∗^	0.96 [0.90 to 1.02]	0.97 [0.93 to 1.02]	0.97 [0.91 to 1.03]	0.95 [0.87 to 1.03]	0.89 [0.80 to 0.99]^∗^

Linear regressions were used for K10 (scale range = 0–40). Ordinal logistic regressions were used for NSSI (scale range = 0–4), suicidal ideation (scale range = 0–2), and suicide attempt (scale range = 0–2). Multivariate models included age (continuous), gender groups, regions, income (4 ordinal levels), and negative healthcare experience index (range = 0–18) or supportive doctor index (range = 0–7).

*P* < 0.05.

*P* < 0.001.

## Discussion

The findings presented in this article provide additional insights into how supportive care from primary care doctors in Aotearoa/New Zealand is associated with lower recent symptoms of psychological distress and lower likelihood of recent suicide attempts, even after controlling for relevant demographics and negative healthcare experiences. These findings replicate findings from the United States about the importance of supportive care^1^ and reiterate the pressing need for primary care doctors to be supportive when providing routine care for transgender people and when discussing gender-affirming care and referral options.^5^

Appropriate transgender knowledge was relatively rare among primary care doctors of transgender people in *Counting Ourselves*, although almost a quarter of participants reported that their primary care doctors knew a lot about gender-affirming care and around half reporting that their primary care doctors have been willing to educate themselves about transgender healthcare. The other half of participants reported feeling required to educate health professionals about transgender people—the most common negative healthcare experience. This finding compares unfavourably to past research in the United States and Sweden,^22–24^ and is double the rate of feeling required to educate health professionals about transgender people in the 2015 US Trans Survey (24.3%).^1,25^ Our findings suggest that the level of training and education that primary care doctors in Aotearoa/New Zealand may fall far below these other countries that Aotearoa/New Zealand often compares itself to; this could be due to Aotearoa/New Zealand being regionally isolated from international standards of best practice in developed in larger countries. Transgender healthcare is not routinely taught in general practice training in Aotearoa/New Zealand, although findings from the *Counting Ourselves* survey are informing changes occurring in the postgraduate GP curriculum to include gender-affirming healthcare going forward. Ensuring all GP trainees are taught the basics of transgender healthcare is essential, and this can be further strengthened by ongoing professional development to ensure particitioners stay up to date. One issue with the continuing professional development programme is that practitioners have choice over which sessions to attend, often resulting in attendees being those who have an existing interest and knowledge in the area rather than upskilling the entire healthcare workforce.

Our findings provide novel evidence that overall supportive experiences with primary care doctors are specifically associated with lower levels of psychological distress and lower likelihood of recent suicidality in Aotearoa/New Zealand. The magnitude of this association was strong, with each unit increase on the Supportive Doctor Index equating to a reduction of 0.50 units in psychological distress on the K10 and OR of 0.89–0.97 for suicidality and NSSI, equating to 3%–11% lower odds of these mental health experiences. The findings also replicate known associations with age and gender.^1,2^ Based on this pattern there are particular groups of transgender people who may require particular support from health professionals. Older transgender participants reported more supportive experiences with primary care doctors and better mental health. This may represent younger transgender participants having worse experiences or less time to experience at least some positive experiences with health professionals. In addition, nonbinary participants reported fewer supportive experiences with primary care doctors but were less likely to report recent suicidal ideation. This may represent transgender people with nonbinary genders being rendered invisible because medical systems often lack the ability to record nonbinary genders.^5^ The mental health of transgender people with binary genders may be more impacted by the long waiting-lists for gender-affirming surgeries in Aotearoa/New Zealand.^32^

Reports of negative healthcare experiences were associated with all 4 mental health variables we analysed, reaffirming known associations between mental health and healthcare experiences in research with transgender people internationally.^1–3^ Each additional negative healthcare experience was associated with a 20% increase in likelihood of having made a suicide attempt, whereas each additional supportive experience with primary care doctors was associated with an 11% decrease in likelihood of having made a suicide attempt. These findings build on past research in the United States showing that experiencing depression or suicidality is less common among transgender people who have health professionals treat them with respect and those who have not needed to educate their health professionals.^1^

Whilst it is of great concern that negative healthcare experiences are associated with such severe distress, we note that participants experienced more than twice the supportive index experiences (3 out of 7) with primary care doctors compared with the negative healthcare index experiences (3 out of 18). By removing barriers to medical student transgender healthcare teaching and improving training for primary care doctors, there is potential to further increase these supportive experiences, which may result in further reductions in psychological distress.^17^

### Strengths and limitations

The sample size relative to the population of Aotearoa/New Zealand and also the diversity of key demographics like age and gender are a strength of the *Counting Ourselves* survey. There was, however, a relatively small proportion of nonbinary participants assigned male at birth, but this is very similar to the gender split in the 2015 US Trans Survey.^1,24^ A small proportion of participants did not complete the section of our survey on general healthcare access due to not having a regular healthcare provider, and it is possible that these people may have worse mental health or worse healthcare experiences.

The *Counting Ourselves* data were collected in 2018 prior to the Covid-19 pandemic. It is possible that restrictions to healthcare access during lockdowns have had a particular impact for transgender people due to sudden changes to how care is delivered and growing waiting-lists for mental healthcare in Aotearoa/New Zealand even though the effects of the pandemic are not as widespread in this country as many others. Gender-affirming surgeries may have been delayed for some transgender individuals or in some regions of Aotearoa/New Zealand.^32^ At the same time, the current government has instigated improved provision of gender-affirming healthcare in Aotearoa/New Zealand,^33^ and further research is needed to track the resulting outcomes.

The *Counting Ourselves* data were gathered cross-sectionally, and our analyses cannot indicate a direction of influence in the association between supportive care and mental health. It is possible that supportive care can lead to better mental health but it is also possible that transgender people with worse mental health may find consultations less supportive if their needs are not met by the limited referral options in Aotearoa/New Zealand. The latest World Professional Association for Transgender Health Standards of Care require that significant mental health concerns be “reasonably well controlled” for people to access gender-affirming hormones and surgeries^34^; this may have resulted in our participants with mental health concerns receiving more gatekeeping and less supportive care. The measures we analysed are limited by focussing on generic experiences, and these did not capture nuance to negative experience and supportive experiences. In addition, the measures were retrospective and had varied periods of recall of 4 weeks or 12 months for mental health or lifetime experiences of negative or supportive healthcare. Future longitudinal studies are needed to provide stronger evidence about temporal precedence of supportive primary and mental health outcomes for transgender people with measures that provide more fine-grained examination of healthcare experiences with different health professionals.

It is also possible that transgender people of different ethnicities, particularly Indigenous peoples like Māori, experience more nuance to healthcare interactions than our measures could detect. Future research could take an in-depth approach by applying qualitative methods and methods from non-Western cultures, such as kaupapa Māori research led by transgender people from the community in question.^35^ Although we measured positive aspects of healthcare, we only recorded negative aspects of mental health (i.e. psychological distress and suicidality). Future research could apply positive frameworks such as flourishing or achieving gender euphoria at the same time as tracking negative mental health outcomes that remain in need of attention.

## Conclusions

The findings of this study reinforce the urgent need to provide high-quality gender-affirming and routine primary care for transgender people. This may help to address the large mental health disparities faced by this population. The relevance of healthcare experiences for mental health builds on recent research demonstrating disparities in Aotearoa/New Zealand and expanding on the association of suicidality with experiences of discrimination and lower levels of general social support.^9,18^ In this study, the findings are specific to the central role of primary care doctors. Our findings also build on existing models of mental health disparities related to cisnormativity associating with negative healthcare experience or lack of supportive experiences for transgender people.^6,36,37^

The provision of healthcare for transgender people is not just about appropriately funded gender-affirming healthcare in an equitable way across Aotearoa/New Zealand and globally, although this is needed.^32^ Our findings support recent calls for enhancements to initial training of health professionals and wide availability of continuing professional development to upskill the existing health workforce,^14,16,17^ so as to address the specific aspects of affirmative care raised by transgender people such as autonomous models of informed consent.^38^ Our findings add to past research demonstrating collaborative processes that can enhance training.^17,18^ Specifically, education is needed that builds transgender competence and confidence in provision of gender-affirming care so as to reduce educational burden from transgender people themselves. The outcomes of improved care for transgender people remains to be seen, but they will hopefully result in improved mental health outcomes and optimization of gender-affirming primary care.

## Data Availability

The data underlying this article cannot be shared publicly as the participants did not agree to this.
